# Proliferation-Stimulating Effect of Colony Stimulating Factor 2 on Porcine Trophectoderm Cells Is Mediated by Activation of Phosphatidylinositol 3-Kinase and Extracellular Signal-Regulated Kinase 1/2 Mitogen-Activated Protein Kinase

**DOI:** 10.1371/journal.pone.0088731

**Published:** 2014-02-10

**Authors:** Wooyoung Jeong, Jinyoung Kim, Fuller W. Bazer, Gwonhwa Song

**Affiliations:** 1 Department of Biotechnology, College of Life Sciences and Biotechnology, Korea University, Seoul, Republic of Korea; 2 Department of Animal Resources Science, Dankook University, Cheonan, Republic of Korea; 3 Center for Animal Biotechnology and Genomics and Department of Animal Science, Texas A & M University, College Station, Texas, United States of America; Michigan State University, United States of America

## Abstract

Colony-stimulating factor 2 (CSF2), also known as granulocyte macrophage colony-stimulating factor, facilitates mammalian embryonic development and implantation. However, biological functions and regulatory mechanisms of action of porcine endometrial CSF2 in peri-implantation events have not been elucidated. The aim of present study was to determine changes in cellular activities induced by CSFs and to access CSF2-induced intracellular signaling in porcine primary trophectoderm (pTr) cells. Differences in expression of *CSF2* mRNA in endometrium from cyclic and pregnant gilts were evaluated. Endometrial *CSF2* mRNA expression increases during the peri-implantation period, Days 10 to 14 of pregnancy, as compared to the estrous cycle. pTr cells obtained in Day 12 of pregnancy were cultured in the presence or absence of CSF2 (20 ng/ml) and LY294002 (20 µM), U0126 (20 µM), rapamycin (20 nM), and SB203580 (20 µM). CSF2 in pTr cell culture medium at 20 ng/ml significantly induced phosphorylation of AKT1, ERK1/2, MTOR, p70RSK and RPS6 protein, but not STAT3 protein. Also, the PI3K specific inhibitor (LY294002) abolished CSF2-induced increases in p-ERK1/2 and p-MTOR proteins, as well as CSF2-induced phosphorylation of AKT1. Changes in proliferation and migration of pTr cells in response to CSF2 were examined in dose- and time-response experiments. CSF2 significantly stimulated pTr cell proliferation and, U0126, rapamycin and LY294002 blocked this CSF2-induced proliferation of pTr cells. Collectively, during the peri-implantation phase of pregnancy in pigs, endometrial CSF2 stimulates proliferation of trophectoderm cells by activation of the PI3K-and ERK1/2 MAPK-dependent MTOR signal transduction cascades.

## Introduction

In pregnant gilts, porcine zygotes undergo cleavage to reach the 4-cell stage when they migrate into the uterine horns and develop to the blastocyst stage and hatch from the zona pellucida by Day 7 of pregnancy. If viable embryos are not present in the uterus, the gilt returns to estrus approximately 21 days after the previous period of estrus. The pig embryo moves from the oviduct into the uterus at about the four-cell stage, i.e., 60 to 72 h after onset of estrus and reaches the blastocyst stage by Day 5. The spherical blastocyst (0.5–1 mm diameter) sheds the zona pellucida between Days 6 and 7 and expands to 2 to 6 mm diameter on Day 10 [1]. On approximately Day 5 after fertilization, blastocyst formation occurs with a distinct trophectoderm and inner cell mass [1]. After hatching from the zona pellucida on Days 6 to 7 of pregnancy, the conceptus transforms into filamentous form and initially secretes estrogens between Days 11 to 12 of pregnancy to signal pregnancy recognition. During this peri-implantation period, blastocysts elongate rapidly from spherical, to tubular and filamentous forms as conceptuses (embryo and its extra-embryonic membranes). This involves various cellular activities including increases in proliferation, migration, and gene expression by trophectoderm cells, as well as extra-embryonic endoderm [2], [3]. In contrast to species with an invasive type of implantation, elongation of conceptuses in pigs with epitheliochorial placenta is critical to establishing sufficient surface area of contact between trophectoderm and uterine epithelia for nutrient and gas exchange and placentation [4]. In general, conceptuses with the most extensive elongation acquire the greatest uterine surface area of contact resulting in the highest chance of survival. The porcine filamentous conceptuse remains free-floating until Days 13 to 14 of pregnancy, and then make initial attachment to the uterine luminal epithelium (LE), and implantation is complete resulting in the interdigitation of uterine LE and trophectoderm in preparation for establishment of a a true epitheliochorial type of placenta by Day 24 of pregnancy [2], [5], [6], [7], [8], [9], [10]. For this process, the conceptus is dependent upon an astonishing amount of secretions from uterine LE and glandular epithelium (GE), that is known collectively as histotroph, which includes hormones, enzymes, nutrients, growth factors, cytokines and other factors [11], [12], [13], [14], [15], [16], [17]. Deficiencies in histotroph-mediated local communication at the maternal-conceptus interface leads to a majority of conceptus mortality and poor outcomes of pregnancy [18], [19].

One key factor, colony-stimulating factor 2 (CSF2), is a cytokine considered essential for the survival, proliferation and differentiation of blood cells such as granulocyte and macrophages [20], [21], [22]. However, CSF2 is also proving to be intimately associated with several diverse aspects of pregnancy physiology including cell-cell adhesion and implantation, and fetal-placental growth and differentiation [23]. Knockout of the CSF2 gene in mice can have subtle effects on early embryonic development and maintenance of pregnancy. For example, mice deficient in CSF2 have blastocysts with reduced numbers of cells which is linked to impaired placental structure and increased mortality of early conceptuses [24], [25].

During the period corresponding to fertilization and the peri-implantation period of pregnancy, CSF2 is expressed primarily by uterine LE and GE in humans and other mammalian species including sheep, cow, and pigs [26], [27], [28], [29], [30], [31], [32]. Through targeting cells of the pre-implantation embryo, CSF2 promotes blastocyst formation and subsequent development, and increases the number of cells in blastocysts in human, mouse, rat, cow and sheep [23], [33], [34]. Supplementation of *in vitro* culture medium with exogenous CSF2 has a beneficial effect on development of the pre-implantation blastocyst in various species [23], [25], [33].

The effects CSF2 are mediated via heterodimeric receptor complexes comprising an α- and a β subunit. The α-subunit of the CSF2 receptor, also known as GM-Rα, binds only CSF2 with low affinity, while the β-subunit is common for interleukin (IL)-5 and IL-3 receptors and converts the low affinity interaction to high affinity binding of ligand [35]. Non-lymphohematopoietic cells such as endothelial cells and placental trophoblast cells exhibit biological responsiveness to CSF2 independent of the β-subunit of the CSF2 receptor [36], [37]. In women and mice, expression of GM-Rα is readily detectable at the time of fertilization of the oocyte and subsequent stages of blastocyst development when it is expressed in both trophectoderm and inner cell mass [25], [38]. The intracellular signaling mediated by the CSF2 receptor has not been studied in porcine conceptus trophectoderm, but experiments using other cell types have described CSF2 cell signaling. The CSF2 receptor transduces signals mainly through the JAK-STAT, MAPK and PI3K dependent pathways to elicit changes in target cell gene expression [39], [40].

There is evidence that CSF2 promotes proliferation of bovine trophectoderm cells prior to and during the peri-implantation period of pregnancy [41]. Also, during the peri-implantation period, time-dependent secretions from trophectoderm, including estrogens, interferons and PGE_2_, stimulate expression of CSF2 by the uterine epithelia [23], [41]. Based on this evidence, porcine CSF2 appears promising as a factor that stimulates conceptus development. However, little is known about CSF2-mediated intracellular signaling pathways in porcine trophectoderm cells and how it stimulates development of the peri-implantation conceptus. Therefore, in the present study, we provide evidence that porcine CSF2 is a factor stimulatory to conceptus development during the peri-implantation period of pregnancy through activation of key cell signaling mechanisms. This study was performed to determine: 1) the temporal expression of *CSF* mRNA in porcine endometrium during the estrous cycle and early pregnancy; 2) effects of CSF2 on transactivation of intracellular signaling pathways in porcine trophectoderm cells; and 3) functional effects of CSF2 on proliferation of pTr cells.

## Results

### Temporal expression of CSF2 mRNA in porcine endometrium during the estrous cycle and pregnancy

**Figure 1:**
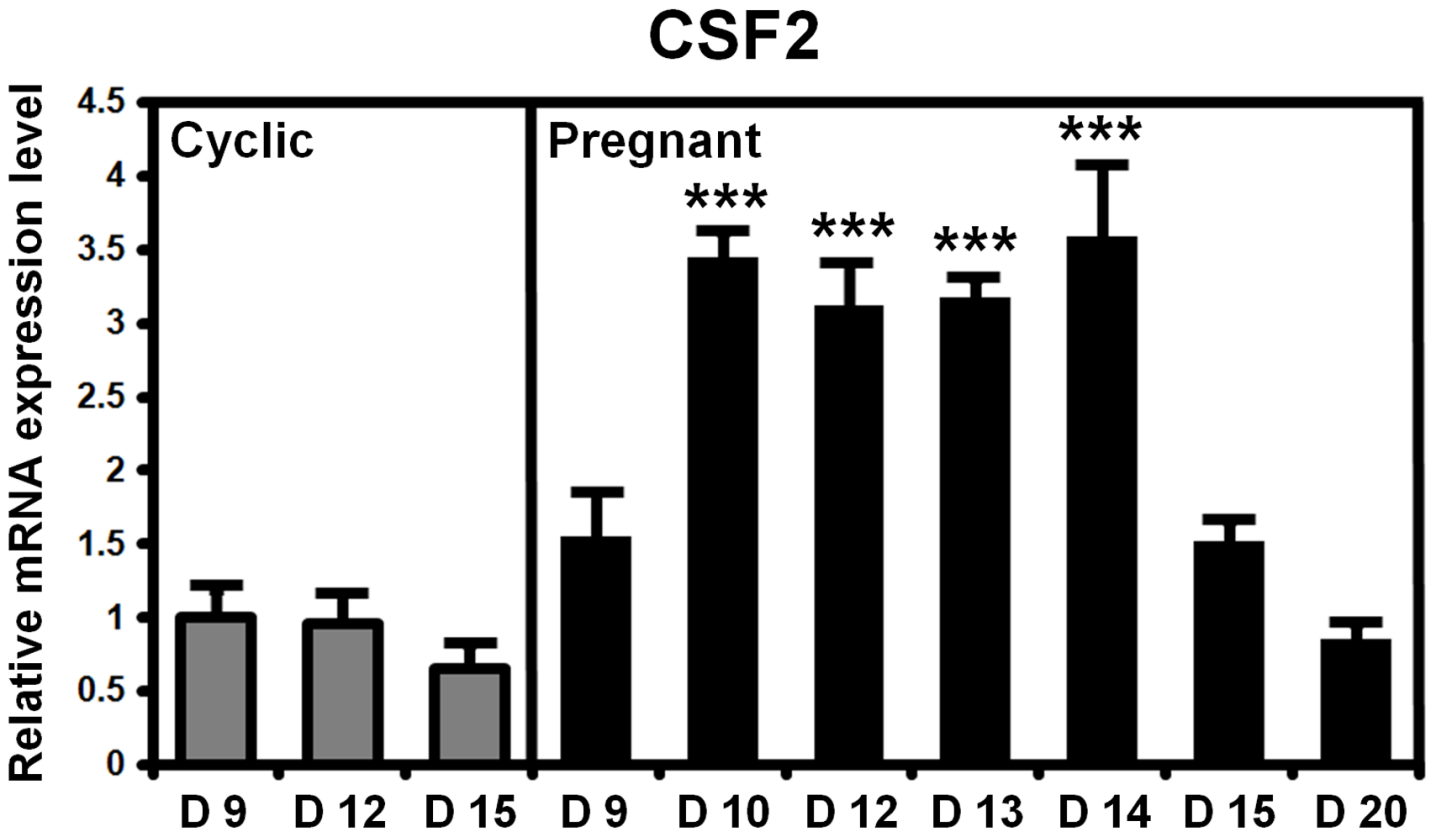
Relative quantification of *CSF2* mRNAs in porcine endometrium during the estrous cycle and pregnancy. Expression levels of endometrial *CSF2* mRNA were not affected by day of the estrous cycle, but effects of day of pregnancy on expression of *CSF2* mRNA were significant (day, *P*<0.01). Endometrial *CSF2* mRNA expression increased between Day 10 and Day 14 of pregnancy. The asterisks denote statistically significant differences (**P<0.01).

Uterine *CSF2* mRNA levels in cyclic and pregnant pigs were determined by quantitative RT-PCR analysis (Figure 1). No significant differences were found in *CSF2* transcripts due to day of the estrous cycle (day, *P*>0.05), but expression of *CSF2* mRNA increased between Days 10 and 14 of pregnancy (*P*<0.001) and then declined to Day 20 of pregnancy. The expression of *CSF2* mRNA on Day 12 of pregnancy was 2.0-fold (*P*<0.05) and 1.9-fold (*P*<0.05) higher than on Days 9 and 15 of pregnancy, respectively, whereas the difference between Days 9 and 15 of pregnancy was less marked (*P*>0.05). Also, expression of *CSF2* mRNA was approximately 3.2-fold (*P*<0.01) and 2.3-fold (*P*<0.01) greater in pregnant than cyclic gilts on Day 12 and Day 15, respectively. But such difference was not significant on Day 9 (*P*>0.05). These results demonstrate that endometrial expression of *CSF2* is up-regulated during peri-implantation period of pregnancy when it is likely involved in peri-implantation events associated with conceptus development.

### CSF2 stimulates proliferation of pTr cells and activates AKT1, ERK1/2, MTOR, p70RSK and RPS6 phosphorylation

**Figure 2:**
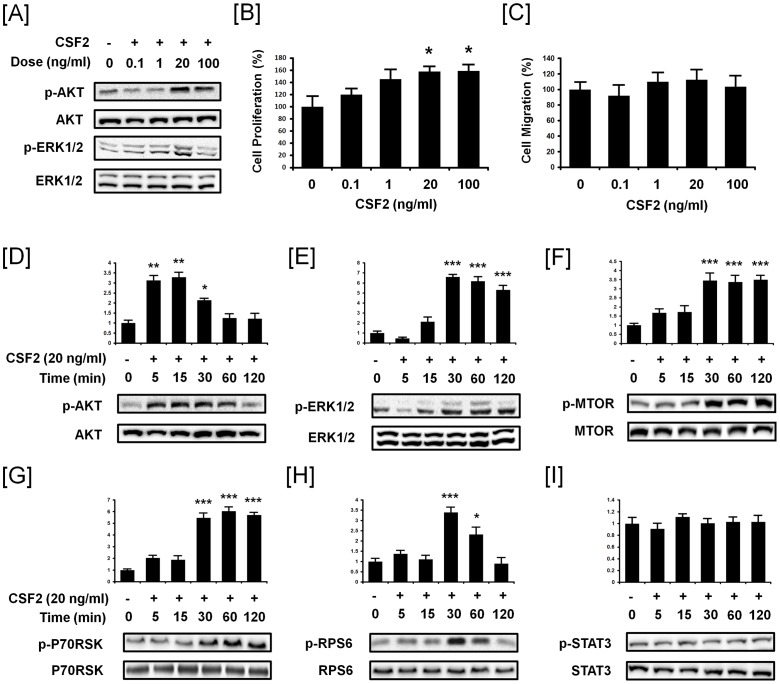
CSF2 increases the abundance of phosphorylated AKT1, ERK1/2, MTOR, p70RSK and RPS6 proteins and stimulates proliferation of pTr cells. [A] Western blot analyses showing dose-dependent effects of CSF2 to increase the abundance of p-AKT1 and p-ERK1/2 proteins in pTr cells *in vitro*. Tubulin-alpha (TUBA) was detected as a total protein control. [B] The effect of CSF2 (0, 0.1, 1, 20 and 100 ng/ml) on pTr cell proliferation. [C] The effect of CSF2 (0, 0.1, 1, 20 and 100 ng/ml) on pTr cell migration. [D-I] Detection of phosphorylated- and total AKT1, ERK1/2, MTOR, p70RSK, RPS6 and STAT3 proteins between 0 and 120 min after treatment of pTr cells with CSF2. Blots were imaged to calculate the normalized values presented in the *graph* by measurements of abundance of phosphorylated proteins relative to total protein. The asterisks denote statistically significant differences (***P<0.001, **P<0.01 and *P<0.05).

To determine the effects of different concentrations of CSF2 (0, 0.1, 1, 20, 100 ng/ml) on signaling events, pTr cells were incubated with CSF2 for 30 min in serum-reduced medium. CSF2 stimulated phosphorylation of AKT1 and ERK1/2 proteins in a dose-dependent manner (Figure 2A). Under the same specific culture conditions, we determined effects of different concentrations of CSF2 on proliferation and migration of pTr cells (Figure 2B and 2C). CSF2 at 20 ng/ml or 100 ng/ml increased pTr cell proliferation by approximately 158% and 159%, respectively (*P*<0.05). On the other hand, there were no significant effects of CSF2 on pTr cell migration. Based on results from our dose-dependent experiments, CSF2 was used at 20 ng/ml in experiments to determine cell signaling pathways involved in pTr cell proliferation. As compared to basal values, CSF2 stimulated a rapid 3.2-fold (*P*<0.01) increase in phosphorylated AKT1 (p-AKT1) within 15 min post-treatment which then gradually decreased to basal levels at 120 min (Figure 2D). Within 30 min after CSF2 treatment, phosphorylated ERK1/2 MAPK (p-ERK1/2 MAPK) increased 6.6-fold (*P*<0.001), and then gradually decreased to 120 min, but remained higher (*P*<0.001) than basal levels (Figure 2E). CSF2 also increased the abundance of phosphorylated MTOR (p-MTOR), p70RSK (p-p70RSK) and RPS6 (p-RPS6) in a time-dependent manner by 3.4-, 5.5- and 3.3-fold (*P*<0.001), respectively, at 30 min post-treatment with only p-RPS6 returning to basal levels by 120 min (Figure 2F, 2G and 2H). On the other hand, there was no significant effect (*P*>0.05) of CSF2 on abundance of phosphorylated STAT3 (p-STAT3) protein in pTr cells (Figure 2I).

### CSF2 activates PI3K- and ERK1/2 MAPK-dependent MTOR signal transduction pathways in pTr cells

**Figure 3:**
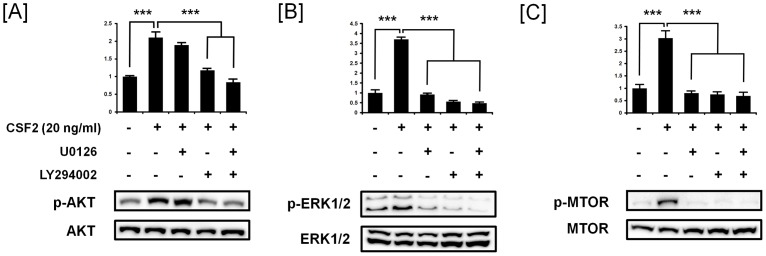
Inhibition of CSF2-induced AKT1 and ERK1/2 phosphorylation in pTr cells. [A] The effects of the PI3K inhibitor (LY294002) and MAPK inhibitor (U0126) on CSF2-induced p-AKT1 proteins. [B] The effects of the LY294002 and U0126 on CSF2-induced p-ERK1/2 proteins. [C] The effects of the LY294002 and U0126 on CSF2-induced p-MTOR proteins. Serum starved pTr cells were incubated with 20 µM LY294002 or 20 µM U0126 for 1 h and then stimulated with CSF2 for 30 min. Blots were imaged to calculate the normalized values presented in the *graph* by measurements of levels phosphorylated proteins relative to total proteins. The asterisk denotes statistically significant differences (***P<0.001).

Effects of CSFs on PI3K-AKT1 and ERK1/2 MAPK cell signal transduction in pTr cells were investigated using pharmacological inhibitors of PI3K (20 µM LY294002) and ERK1/2 MAPK activity (20 µM U0126). Serum starved pTr cells were incubated with either LY294002 (20 µM) or U0126 (20 µM) 1 h prior to treatment with CSF2 (20 ng/ml). CSF2-induced phosphorylation of AKT1 was inhibited completely by the PI3K inhibitor (*P*<0.001), while U0126 did not affect the abundance of CSF2-induced p-AKT1 protein (*P*>0.05) (Figure 3A). CSF2-induced increases in phospho-ERK1/2 were blocked by both U0126 (*P*<0.001) and LY294002 (*P*<0.001) (Figure 3B). Also, CSF2-induced phospho-MTOR proteins were less abundant (P<0.001) in pTr cells treated with both U0126 (*P*<0.001) and LY294002 (*P*<0.001) (Figure 3C). These results suggest that PI3K is upstream of the ERK1/2 MAPK pathway and that MTOR is a common downstream target of these parallel cell signaling pathways induced by CSF2 in pTr cells.

### CSF2 induced stimulation of pTr cell proliferation is mediated via the PI3K-MTOR and ERK1/2 MAPK cell signaling pathways

**Figure 4:**
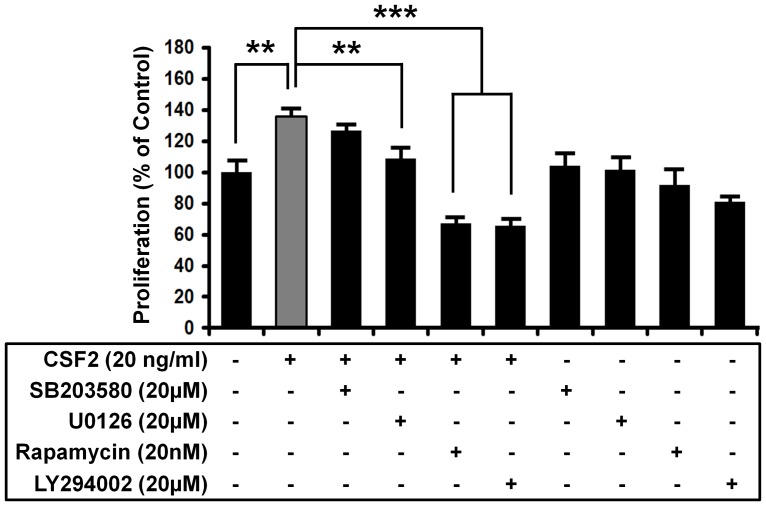
CSF2 stimulates proliferation of pTr cells through PI3K-MTOR and ERK1/2 MAPK pathways. The pTr cells seeded in a 96-well microplate were treated with recombinant CSF2 (20 ng/ml), SB203580 (20 µM), U0126 (20 µM), rapamycin (20 nM), LY294002 (20 µM), or their combination for 24 h in serum-free medium. After labeling of cells with BrdU for 24 h, cell numbers were determined by measuring the absorbance values of the reaction product. Data are expressed as a percentage relative to non-treated control pTr cells (100%). CSF2 stimulated (*P*<0.01) pTr cell proliferation and, U0126 (20 µM), rapamycin (20 nM) and LY294002 (20 µM) blocked (*P*<0.001 or *P*<0.01) this CSF2-induced effect. However, there was no significant inhibitory effect of CSF2 by the inhibitor of P38 MAPK activity (SB203580). The asterisks denote a significant effect of CSF2 treatment (***P<0.001 and **P<0.01).

To confirm whether PI3K-AKT1 and ERK1/2 MAPK signal transduction pathways are involved in CSF2-induced stimulation of pTr cell proliferation, cell proliferation assays were conducted in the presence or absence of CSF2 (20 ng/ml), U0126 and LY294002. As shown in Figure 4, CSF2 increased pTr cell proliferation by approximately 139% (*P*<0.01). However, pretreatment with either U0126 (*P*<0.001) or LY294002 (*P*<0.01) inhibited CSF2-induced proliferation of pTr cells as compared to effects of CSF2 alone. Pharmacological inhibitors of FRAP/mTOR activity (rapamycin) also reduced effects of CSF2 on proliferation of pTr cells (*P*<0.001). To examine involvement of other intracellular messengers, we used a pharmacological inhibitor of P38 MAPK (SB203580) to find that it did not inhibit proliferation of pTr cells in response to CSF2 (*P*>0.05). These results suggest that activation of PI3K-AKT1-MTOR and ERK1/2 MAPK signaling pathways play a central role in stimulation of pTr cell proliferation in response to uterine-derived CSF2 during the peri-implantation period of pregnancy.

## Discussion

Results of the present study indicate a key role for CSF2 in stimulating proliferation of pTr cells via the PI3K-AKT1-dependent ERK1/2 MAPK cell signaling cascade. This novel finding supports our hypothesis that during the peri-implantation period of pregnancy in pigs, CSF2 from uterine endometrium stimulates growth and development of the conceptus. Establishment of molecular crosstalk between mother and conceptus is most important for establishment and maintenance of pregnancy in the pig because survival and development of the conceptus depends on histotroph in the uterine lumen for nutrients, growth factors and cytokines [2], [16], [42]. CSF2 is implicated as an embryotrophic factor underpinning embryogenesis and implantation in most mammalian species.

There is emerging evidence that CSF2 improves the developmental rate of *in vitro* cultured embryos by increasing cell numbers in blastocysts [33], [43]. Human embryos cultured with CSF2 reached the blastocyst stage earlier and had greater numbers of cell, which correlated with successful establishment of pregnancy after human IVF and embryo transfer [34], [44]. Studies of CSF2 null mice demonstrated that they have fewer cell numbers in the inner cell mass at the blastocyst stage [25]. Also, addition of CSF to embryo culture medium rescued deficiencies in placental structure and fetal growth, and also improved postnatal development of mouse pups [25], [45]. Effects of CSF2 to stimulate *in vitro* development of embryos have also been reported for bovine and ovine embryos [28], [46]. Growth and survival of bovine and ovine conceptuses in vitro was also promoted by CSF2 [47], [48], [49].

The embryotrophic effects of CSF2 are likely to be more profound in pigs which experience high rates of embryonic mortality, and highly variable rates in conceptus development and implantation [34]. During the peri-implantation period of pregnancy, porcine conceptuses elongate rapidly and trophectoderm then attaches to uterine LE which is especially important to ensure that pig conceptuses maximize contact with uterine endometrial surface for the efficient exchange of nutrients and gases [2], [5], [6], [7], [8], [18], [50], [51]. Despite the requirement for histotrophic to induce active cell proliferation and differentiation during this period, previous studies with pigs have focused mainly on effects of CSF2 on development during the cleavage stages of embryonic development and not the peri-implantation phase when histotroph can have dramatic effects on conceptus development and survival [30]. Therefore, the present study addressed the unresolved issue of effects of CSF2 on conceptuses during the peri-implantation period of pregnancy.

Throughout pregnancy, *CSF2* mRNA is expressed in the uterine endometrium, especially uterine LE and GE in species such as human, mouse, cow and sheep [26], [28], [52], [53], [54]. In the human and mouse, CSF2 synthesis remains high for the first few days following fertilization in response to estrogen, and then declines at the time of implantation of the blastocyst when the uterus is under the influence of progesterone [55], [56]. But, little information is available on temporal variations in expression of CSF2 in the uterus during early pregnancy and the estrus cycle. In the present study, we demonstrated temporal changes in expression of the *CSF2* mRNA in the porcine endometrium on different days of the estrous cycle and early pregnancy. Our result revealed that *CSF2* mRNA expression significantly increases between Days 10 and 14 of pregnancy, and that expression levels on Days 12 and 15 of pregnancy are higher than those on the same days of the estrous cycle. This expression pattern is likely related to stimulatory effects of conceptus-derived estrogens during the peri-implantation phase of pregnancy. As well as increased secretion of CSF2 in response to estrogen, expression of CSF2 in cows is also stimulated by both conceptus derived-interferon tau (IFNT) and PGE2 before and after the attachment phase of implantation [41], [47], [48], [49], [52]. Similarly, in sheep, uterine-derived CSF2 has been reported to stimulate secretion of IFNT by ovine trophoblast and enhance uterine support for conceptus development during the period of pregnancy establishment [28]. Results of the present study indicate that CSF2 plays an essential role in peri-implantation events via supporting the development and survival of the porcine conceptus and this may include stimulation of secretion of numerous factors from trophectoderm including interferon delta.

In the present study, we determine that CSF2-induced cell signaling cascades in pTr cells that increased cell proliferation. Although numerous studies have shown that CSF2 receptors are expressed in early embryos, knowledge of downstream signaling cascades triggered by CSF2 in developing embryos is limited. In other cell types, CSF2 activates several cell signaling pathways, including the PI3K-AKT, JAK-STAT and MAPK cascades via interaction with the β subunit of the CSF2 receptor [57]. In the present study, time-dependent increases in phosphorylation of AKT1, ERK1/2, MTOR and p70RSK were detected in CSF2 treated pTr cells. The MTOR-RPS6K cell signaling pathway plays central roles in various cellular activities such as cell proliferation, differentiation, and protein synthesis [58]. Recently, we reported that several amino acids including arginine, leucine, and glutamine stimulate pTr cell proliferation through activation of the MTOR-RP6K-RPS6 signaling pathway [59]. In response to CSF2, the activated PI3K-MTOR-p70RSK cascades likely transduces signaling through downstream pathways such as RPS6K-RPS6-EIF4EBP1, leading to mRNA translation and protein synthesis required for cell proliferation and growth [60], [61]. ERK1/2 MAPK has been implicated as a major participant in proliferation and differentiation processes, including embryonic and placental development in a number of model systems [62], [63], [64], [65]. Along with the RTK-MAPK pathway, the JAK-STAT cell signaling pathway often mediates effects of numerous cytokines which influence migration, proliferation and differentiation of trophoblast cells [66]. Furthermore, CSF2 can stimulate the JAK-STAT signaling pathway through tyrosine phosphorylation of STAT3 that is indispensable for embryogenesis and invasion of human transformed trophoblast cells [66], [67], [68]. However, in the present study, there was no significant effect of CSF2 on phosphorylated STAT3 proteins in pTr cells. Taken together, our results suggest that activation of both PI3K-AKT1-MTOR and ERK1/2 MAPK cell signaling cascades are induced simultaneously by CSF2 in pTr cells.

We also found that the ERK1/2 MAPK specific inhibitor (U0126) inhibited CSF2-induced activation of ERK1/2 and MTOR, but had no significant effect on AKT1 activation. However, the PI3K specific inhibitor (LY294002) inhibited CSF2-induced phosphorylation of ERK1/2 and MTOR as well as CSF2-induced phosphorylation of AKT1. These results indicate that ERK1/2 MAPK cell signaling is activated by the PI3K-AKT1 pathway in response to CSF2 and that MTOR protein is a common downstream target for phosphorylation and for cross-talk between these two cell signaling cascades. Furthermore, we demonstrate that pharmacological inhibitors of ERK1/2, PI3K and FRAP/mTOR activity (rapamycin) abolished CSF2-induced pTr cell proliferation. But, there was no significant effect of inhibiting P38 MAPK activity (SB203580) on effects of CSF2-induced proliferation of pTr cells. Taken together, these results suggest that CSF2 has stimulatory effects on proliferation of pTr cells that are mediated via activation of PI3K- and ERK1/2 dependent MTOR cell signaling pathways. CSF2 deficient mice exhibit reduced placental development due to reduced proliferation of mononuclear trophoblast cells [69]. This CSF2-induced proliferation of mononuclear trophoblast cells is likely accompanied by enhanced glucose uptake and/or anti-apoptotic processes [25], [70]. When mouse embryos are exposed to stressors, CSF2 is likely to exert protective effects against cytotoxic mechanisms, with increased expression of anti-apoptotic factors such as Bcl-2 [25], [70], [71], [72].

In conclusion, endometrial CSF2 stimulates proliferation of porcine conceptus trophectoderm in a paracrine manner during the peri-implantation period. This beneficial event occurs via PI3K-dependent MTOR and MAPK signal transduction cascades. Results of the current study provide important insights into CSF2-induced regulatory mechanisms for successful development and implantation of the porcine conceptus, and, this could assist in improving survival of conceptuses during the peri-implantation period of pregnancy.

## Materials and Methods

### Experimental Animals and Animal Care

Sexually mature gilts of similar age, weight, and genetic background were observed daily for estrus (Day 0) and exhibited at least two estrous cycles of normal duration (18–21 days) before being used in this study. All experimental and surgical procedures were in compliance with the Guide for Care and Use of Agricultural Animals in Teaching and Research and approved by the Institutional Animal Care and Use Committee of Texas A&M University.

### Experimental Design and Tissue Collection

Gilts were assigned randomly to either cyclic or pregnant status. Those in the pregnant group were bred when detected in estrus and 12 and 24 h later. Gilts were ovariohysterectomized on either Day 9, 12, or 15 of the estrous cycle or on Day 9, 10, 12, 13, 14, 15 or 20 of pregnancy (n = 3–4 pigs per day per status). For confirmation of pregnancy prior to implantation, the lumen of each uterine horn was flushed with 20 ml of physiological saline and examined for the presence of morphologically normal conceptuses. Uteri from cyclic and pregnant gilts were processed to obtain several sections (∼0.5 cm) from the entire uterine wall in the middle of each uterine horn. The tissue was fixed in fresh 4% paraformaldehyde in PBS (pH 7.2) and embed the tissue in Paraplast-Plus (Oxford Laboratory, St. Louis, MO) as described previously [7].

### Cell culture

Mononuclear porcine trophectoderm (pTr) cells from Day 12 pig conceptuses were cultured and used in the present *in vitro* studies as described previously [73], [74]. For experiments, monolayer cultures of pTr cells were grown in culture medium to 80% confluence in 100-mm tissue culture dishes. Cells were serum starved for 24 h, and then treated with recombinant porcine CSF2 (20 ng/ml; R&D Systems, Inc., Minneapolis, MN) for 0, 5, 15, 30, 60 or 120 min. Based on preliminary dose-response experiments, 20 ng/ml CSF2 was selected for use in all experiments in the present study. This design was replicated in three independent experiments.

### RNA Isolation

Total cellular RNA was isolated from endometrium from cyclic and pregnant gilts using Trizol reagent (Invitrogen, Carlsbad, CA) and purified using an RNeasy Mini Kit (Qiagen) according to the manufacturer's recommendations. The quantity and quality of total RNA was determined by spectrometry and denaturing agarose gel electrophoresis, respectively.

### Quantitative PCR Analysis

Specific primers for porcine *CSF2* (forward: 5′- TGT TGG CCA AGC ACT ATG AG -3′; reverse: 5′- CAA AGG GGA TGG TGA AAA GA -3′) were designed from sequences in the GenBank data base using Primer 3 (ver.4.0.0). All primers were synthesized by Bioneer Inc.(Daejeon, Korea). Gene expression levels were measured using SYBR® Green (Sigma, St. Louis, MO, USA) and a StepOnePlus™ Real-Time PCR System (Applied Biosystems, Foster City, CA, USA). The PCR conditions were 95°C for 3 min, followed by 40 cycles at 95°C for 20 sec, 64°C for 40 sec, and 72°C for 1 min using a melting curve program (increasing the temperature from 55°C to 95°C at 0.5°C per 10 sec) and continuous fluorescence measurements. Sequence-specific products were identified by generating a melting curve in which the C_T_ value represented the cycle number at which a fluorescent signal was significantly greater than background, and relative gene expression was quantified using the 2^−ΔΔCT^ method. The glyceraldehydes-3-phosphate dehydrogenase (*GAPDH*) gene was used as the endogenous control to standardize the amount of RNA in each reaction.

### Western Blot Analyses

Concentrations of protein in whole-cell extracts were determined using the Bradford protein assay (Bio-Rad, Hercules, CA) with bovine serum albumin (BSA) as the standard. Proteins were denatured, separated using SDS-PAGE and transferred to nitrocellulose. Blots were developed using enhanced chemiluminescence detection (SuperSignal West Pico, Pierce, Rockford, IL) and quantified by measuring the intensity of light emitted from correctly sized bands under ultraviolet light using a ChemiDoc EQ system and Quantity One software (Bio-Rad, Hercules, CA). Immunoreactive proteins were detected using rabbit anti-mouse polyclonal antibodies against p-AKT1 and AKT1 at a 1∶1000 dilution and 10% SDS/PAGE gel; rabbit anti-human polyclonal antibodies against p-MTOR and rabbit anti-human monoclonal antibodies against MTOR at a 1∶1000 dilution and 8% SDS/PAGE gel; rabbit anti-human polyclonal antibodies against p-p70RSK and p70RSK at a 1∶1000 dilution and 10% SDS/PAGE gel; rabbit anti-human polyclonal p-ERK1/2 MAPK and rabbit anti-human monoclonal ERK1/2 MAPK IgG, each at a 1∶1000 dilution, and 12% SDS/PAGE gel. As a loading control, mouse anti-α-tubulin (TUBA) was used to normalize results from detection of proteins by western blotting. All antibodies were from Cell Signaling Technology. Multiple exposures of each western blot were performed to ensure linearity of chemiluminescent signals.

### Proliferation assay

Proliferation assays were conducted using Cell Proliferation ELISA, BrdU kit (Roche) according to the manufacturer's recommendations. Briefly, pTr cells were seeded in a 96-well microplate (tissue culture grade, flat bottom), and then incubated for 24 h in serum-free DMEM/F-12. Cells were then treated with recombinant porcine CSF2 protein and various treatments in a final volume of 100 µl/well. After 24 h of incubation, 10 µM BrdU was added to the cell culture and the cells incubated for an additional 24 h at 37°C. After labeling cells with BrdU, the fixed cells were incubated with anti-BrdU-POD working solution for 90 min. The anti-BrdU-POD binds to BrdU incorporated into newly synthesized cellular DNA and these immune complexes were detected by the reaction to TMB (tetramethyl-benzidine) substrate solution. The absorbance values of the reaction product were quantified by measuring the absorbance at 370 nm using an ELISA reader (Bio-Rad, Seoul).

### Migration assay

The pTr cells (50,000 cells per 100 µl serum and insulin-free DMEM) were seeded on 8-µm pore Transwell inserts (Corning Costar no. 3422; Corning, Inc., Corning, NY). Cells were then treated with recombinant porcine CSF2 protein (0, 0.1, 1, 20 and 100 ng/ml)(n = 3 wells per treatment). After 12 h, cells on the upper side of the inserts were removed with a cotton swab. For evaluation of cells that migrated onto the lower surface, inserts were fixed in 50% ethanol for 5 min. The transwell membranes were then removed, placed on a glass slide with the side containing cells facing up, overlaid with prolong antifade mounting reagent with 4′,6- diamidino-2-phenylindole, and overlaid with a coverslip (Invitrogen- Molecular Probes, Eugene, OR). Migrated cells were counted systematically in five non-overlapping locations, which covered approximately 70% of the insert membrane growth area, using a Zeiss Axioplan 2 fluorescence microscope with Axiocam HR digital camera and Axiovision 4.3 software (Carl Zeiss Microimaging, Thornwood, NY). The entire experiment was repeated at least three times.

### Statistical Analyses

All quantitative data were subjected to least squares ANOVA using the General Linear Model procedures of the Statistical Analysis System (SAS Institute Inc., Cary, NC). Western blot data were corrected for differences in sample loading using the TUBA data as a covariate. All tests of significance were performed using the appropriate error terms according to the expectation of the mean squares for error. A *P* value less than or equal to 0.05 was considered significant. Data are presented as least-square means (LSMs) with SEs.
